# Development of a Simple Pretreatment Immunoassay Based on an Organic Solvent-Tolerant Nanobody for the Detection of Carbofuran in Vegetable and Fruit Samples

**DOI:** 10.3390/biom9100576

**Published:** 2019-10-07

**Authors:** Jin-ru Zhang, Yu Wang, Jie-xian Dong, Jin-yi Yang, Yu-qi Zhang, Feng Wang, Rui Si, Zhen-lin Xu, Hong Wang, Zhi-li Xiao, Yu-dong Shen

**Affiliations:** 1 Guangdong Provincial Key Laboratory of Food Quality and Safety, National-Local Joint Engineering Research Center for Processing and Safety Control of Livestock and Poultry Products, College of Food Science, South China Agricultural University, Guangzhou 510642, China; lovezjr0815@163.com (J.-r.Z.); yjy361@163.com (J.-y.Y.); timzhang@stu.scau.edu.cn (Y.-q.Z.); wangfg024@163.com (F.W.); huanongxiaosi@163.com (R.S.); scau_xzl@163.com (Z.-l.X.); shenyudong@scau.edu.cn (Y.-d.S.); 2 Guangzhou Institute of Food Inspection, Guangzhou 510080, China; xxwangyu@163.com; 3 Department of Entomology and Nematology and UCD Comprehensive Cancer Center, University of California, Davis, CA 95616, USA; jxdong@ucdavis.edu; 4 Neurobiology, Physiology & Behavior, University of California, Davis, CA 95616, USA

**Keywords:** carbofuran, nanobody, enzyme-linked immunosorbent assay

## Abstract

Nanobodies are one-tenth the size of conventional antibodies and are naturally obtained from the atypical heavy-chain-only antibodies present in camelids. Their small size, high solubility, high stability, and strong resilience to organic solvents facilitate their use as novel analytical reagents in immunochemistry. In this study, specific nanobodies against pesticide carbofuran were isolated and characterized from an immunized library via phage display platform. We further established an indirect competitive enzyme-linked immunosorbent assay (ELISA) using nanobody Nb316 to detect carbofuran in vegetable and fruit samples. The results showed a half-maximal inhibitory concentration (IC_50_) of 7.27 ng/mL and a detection limit of 0.65 ng/mL. A simplified sample pretreatment procedure omitting the evaporation of organic solvent was used. The averaged recovery rate of spiked samples ranged between 82.3% and 103.9%, which correlated with that of standard UPLC–MS/MS method. In conclusion, a nanobody with high specificity for carbofuran was characterized, and a nanobody-based sensitive immunoassay for simple and rapid detection of carbofuran in real samples was validated.

## 1. Introduction

Carbofuran is a broad-spectrum insecticide widely used in a variety of agricultural crops to shorten the crop growth period and increase crop yields [1]. Due to its high solubility, carbofuran is easily absorbed by plant roots and transported to various plant organs, especially the edge of leaves [2]. However, carbofuran is highly toxic [3], and its use in agricultural products poses a potential hazard for human health and the environment [4]. The maximum residue limit (MRL) for carbofuran has been established under different legislations worldwide. For example, in the case of Chinese cabbages, cucumbers, and oranges, the MRL is 0.02 mg/kg in China. Therefore, it is necessary to detect carbofuran with high sensitivity and strong specificity.

In recent years, many methods for the detection of carbofuran residues have been developed, such as gas chromatography, high-performance liquid chromatography, fluorescence detection, and immunochemical methods [5,6,7,8,9]. Enzyme-linked immunosorbent assay (ELISA) is a rapid technique for the detection of pesticide residues because of its sensitivity and high-throughput screening ability [10]. Most of the studies used polyclonal or monoclonal antibodies to develop ELISAs.

In 1993, Hamers-Casterman et al. identified naturally occurring heavy-chain-only antibodies in camelids, with variable regions representing antigen-binding pockets [11,12]. Single variable domains of heavy chain antibodies, also known as nanobodies, can be easily obtained from phage display libraries, which are not affected by the heavy/light chain shuffling [13]. Nanobody is advantageous compared with other recombinant antibody fragments in terms of high thermal stability, strong resilience against organic solvents, ease of genetic manipulation, and ease of expression in various expression systems, and is therefore an emerging reagent for use in immunoassay [14,15]. Nanobody-based ELISA methods for the detection of low-molecular-weight contaminants, such as aflatoxin B1 [16], microcystin-LR [17], triazophos [18], parathion [19], fipronil [20], and carbaryl [21] have been published. Carbofuran is an organic chemical, and most of the reported immunoassays involve complex pretreatment (extraction by organic solvent and blown dry by nitrogen). Due to the high organic solvent resistance, nanobody-based immunoassays facilitate rapid and simple monitoring of pesticides in vegetables and fruit samples. 

In this study, we aimed to isolate nanobodies specifically recognize carbofuran from a Bactrian camel (*Camelus bactrianus*) immunized library. The nanobodies were carefully characterized and the optimal nanobody was selected to develop a competitive indirect ELISA. Finally, the presented immunoassay results were validated via comparison with UPLC–MS/MS analysis to spike vegetable and fruit samples.

## 2. Materials and Methods

### 2.1. Materials and Reagents

The haptens 4-[[(2,3-dihydro-2,2-dimethyl-7-benzofuranyloxy) carbonyl]-amino] butanoic acid (BFNB) was previously synthesized in the laboratory [22]. Carbofuran pesticide and its structural analogues were purchased from Hua Xin Bio. Co. Ltd. (Tianjin, China). Keyhole limpet hemocyanin (KLH), ovalbumin (OVA), and Freund’s adjuvant were obtained from Sigma-Aldrich (St. Louis, MO, USA). The TRIzol reagent was purchased from Thermo Fisher Scientific (Shanghai, China). The first strand cDNA synthesis kit was obtained from TaKaRa (Dalian, China). Gel extraction and PCR purification kits were purchased from QIAGEN (Dusseldorf, Germany). Helper phage M13K07, SfiI restriction enzymes, and T4 DNA ligase were purchased from New England Biolabs (Beijing, China). The anti-HA tag antibody (HRP) was obtained from NOVUS Biologicals (Shanghai, China). Primary secondary amine (PSA), graphitized carbon black (GCB), C18 sorbents, and PestiCarb (PC) were purchased from Biocomma Limited (Shenzhen, China).

### 2.2. Construction of Phage Display Nanobody Library

A three-year-old Bactrian camel was immunized subcutaneously with 500 μg of BFNB-KLH and Freund’s adjuvant mixture biweekly. The immunogen BFNB-KLH and coating antigen BFNB-OVA were prepared using the active ester method. One week after the fifth immunization, 100 mL of peripheral blood was collected to complete lymphocyte isolation and total RNA extraction. The cDNA was synthesized using the TaKaRa First Strand RT-PCR Kit. The *VHH* genes were amplified by two-step nested PCR using the following primers: CALL001 (5′GTCCTGGCTGCTCTTCTACAAGG-3′) and CALL002 (5′-GGTACGTGCTG TTGAACTGTTCC-3′) for the first step; Sfi-Fr1 (5′-ACTGGCCCAGGCGGCCGAGGTGCAGCTGSWGSAKTCKG-3′) and Sfi-Fr4 (5′-ACTGGCCGGCCTGGCCTGAGGAGACGGTGACCWGGGTC-3′) in the second step [23]. The *VHH* genes were ligated into the pComb3Xss phagemid vector and then electroporated into the competent ER2738 cells. All cells were cultured on LB plates (containing 100 μg/mL ampicillin and 50 μg/mL tetracycline) overnight and then collected. After the infection of helper phage M13K07, the phage library was precipitated with PEG8000/NaCl (2.5 M NaCl, 25 mM PEG8000) and filtered through a 0.22 µm membrane.

### 2.3. Selection and Identification of Anti-Carbofuran Phage Clones

The library was subjected to four rounds of panning on 96-well microtiter plates. For the first round, two wells of ELISA plate were coated with 10 μg/mL BFNB-OVA antigen (100 μL each) in PBS (137 mM NaCl, 2.7 mM KCl, 4.3 mM Na_2_HPO_4_, and 1.4 mM KH_2_PO_4_) via overnight incubation at 37 °C. Next day, the wells were blocked with 3% BSA for 2 h at 37 °C. The phage library was depleted with 2% KLH, BSA, and OVA, and incubated at 37 °C for 1 h. The unbound phage was transferred to the BFNB-OVA well (100 μL per well) and shaken for 1 h at 37 °C. After washing the plate five times with PBST (PBS containing 0.5% Tween) and 10 times with PBS, the bound phages were competitively eluted with 2 μg/mL carbofuran solution in PBS (100 μL per well) for 1 h shaken at 37 °C. Eluates (10 μL each) were diluted to calculate the panning output titer by plating on LB (100 mg/mL ampicillin, 50 mg/mL tetracycline), and 180 μL of remaining samples were amplified for the next round of panning. A total of four rounds of panning were carried out. For the second, third, and fourth round, the plate was coated with BFNB-OVA at 5, 1, 0.2 μg/mL and the concentration of carbofuran for competitive elution was 1, 0.5, 0.25 ng/mL, respectively. After washing 10 times with PBST each round, the wells in second and fourth round were blocked with 1% gelatin instead of 3% BSA.

To determine the binding activity of clones against carbofuran, 190 clones were selected from the output plates in the third and fourth rounds, and induced by IPTG in deep well plates with LB medium containing 100 μg/mL ampicillin. The supernatant medium was used for indirect competitive ELISA detection after centrifugation at 3000 rpm for 20 min. ELISA plate were coated with 1 μg/mL BFNB-OVA antigen (100 μL each) and were blocked with 3% BSA. All clones with significant inhibition rates (with 1 μg/mL carbofuran) clones were selected as positive clones and sequenced.

### 2.4. Expression and Purification of Nanobody Protein

The plasmid that specifically recognizes carbofuran was extracted from the ER2738 clone and was transformed into BL21(DE3)-competent cells by heat shock (42 °C, 90 s). After sequencing and identification, a single clone was picked and grown in 10 mL of LB medium (100 mg/mL ampicillin) overnight. The next day, 10 mL of the overnight culture was added to 1 L of LB (100 mg/mL ampicillin) and shaken until the OD_600_ reached 0.6–0.8. IPTG was added at a final concentration of 1 mM to induce the expression of nanobody protein with shaking at 250 rpm overnight at 37 °C. Cell pellets were harvested after centrifugation at 12,000× *g* for 20 min. The soluble nanobody protein was isolated from the cells via freezing and thawing method and sucrose osmotic pressure method (destroy cell wall with high osmotic pressure solution (300 mM Tris, 0.65 mM EDTA, 0.5 M sucrose)), and purified using a gravity column packed with 1 mL of Ni-NTA resin [24]. The nanobody proteins were obtained via elution with imidazole using an increasing concentration gradient (10, 20, and 50 mM), and dialyzed five times with PBS. After purification, the nanobody protein was characterized via SDS-PAGE and Western blot (anti-HA tag antibody (HRP)), and the concentration was determined using a NanoDrop 2000C system.

### 2.5. Stability Analysis of Anti-Carbofuran Nanobody

The stability of the nanobody at different temperatures was evaluated. The nanobody was diluted to the working concentration (4 μg/mL) and divided into seven equal portions. It was transferred to a water bath at 20, 35, 50, 65, 80, and 95 °C for 5 min. It was also placed in a 95 °C water bath, and heated for 10, 20, 30, 40, 50, and 60 min. The binding activity of the nanobody to the antigen was measured by ELISA. The activity of the unheated nanobody to bind to the antigen was taken as 100%.

The tolerance of the nanobody to different organic solvents and the same organic solvent at different concentrations was evaluated. Methanol and acetonitrile are the common solvents used for carbofuran extraction and were therefore selected to evaluate the organic solvent tolerance of nanobodies. The nanobody was diluted to the same working concentration using different solvent solutions with different concentrations (10%, 20%, 40%, 60%, and 80%) of methanol and acetonitrile. The binding activity of the antibody to the antigen was determined by ELISA. The antigen-binding activity of the antibody diluted without the organic solvent to bind to the antigen was 100%. 

### 2.6. Nanobody-Based Indirect Competitive ELISA

A 96-well plate (100 μL per well) with 1 μg/mL of BFNB-OVA in PBS was coated by overnight incubation at 37 °C. The next day, the plate was blocked with 1% fish collagen (120 μL per well) at 37 °C for 2 h. Each well was treated with 50 μL of a gradient dilution of carbofuran and 50 μL of 4 μg/mL nanobody. The plate was incubated at 37 °C for 1 h. The plate was washed five times with PBST and exposed to 100 μL of anti-HA-HRP antibody in PBS followed by incubation for 40 min at 37 °C. After washing the plate five times, 100 μL of TMB (3,3′,5,5′-Tetramethylbenzidine) chromogenic solution was added, and the reaction was terminated after 10 min by adding 50 μL of 10% H_2_SO_4_. The absorbance at 450 nm was measured using a microplate reader, and the standard curve was fitted with a four-parameter fitting module of Origin 9.0. The half maximal inhibitory concentration (IC_50_) and limit of detection (LOD) denote the carbofuran concentrations at 50% and 10% inhibition, respectively. The cross-reaction rate was calculated as follows: CR (%) = IC_50_ (carbofuran, ng/mL)/IC_50_ (carbofuran analogues, ng/mL) × 100.

### 2.7. Immunoassay Validation

Samples of fresh Chinese cabbage, cucumber, and orange were obtained from the local market. The samples are simply cleaned and smashed with a homogenizer. The quick, easy, cheap, effective, rugged, and safe (Quechers) EN15662 method was used to pretreat the samples [25]. Briefly, 10 g of a finely crushed vegetable sample (accurate to 0.01) was weighed in a tube and mixed with a standard solution of 0.1 mL (10, 20, and 50 ng/g) carbofuran pesticide prepared using methanol. After the addition of 10 mL acetonitrile, the tube was vortexed for 1 min. An extract salt pack (containing 4 g magnesium sulfate, 1 g sodium chloride, 1 g sodium citrate, and 0.5 g disodium hydrogen citrate) was added, shaken for 1 min, and centrifuged at 4000 rpm for 5 min. A 6 mL aliquot of the supernatant was transferred into a clean tube containing 150 mg PSA, 45 mg GCB, and 900 mg magnesium sulfate, vortexed for 1 min, and centrifuged at 4000 rpm for 5 min. To eliminate the matrix effect, the supernatant was directly diluted five times with PBS buffer and passed through a 0.22 μm filter to obtain the sample. The carbofuran content and addition recovery were determined using the indirect competitive ELISA (ic-ELlSA). The recovery rate was expected to remain within a relatively stable and narrow interval (80%–120%). The addition recovery rate (R, %) and the coefficient of variation (CV, %) were calculated according to the following formula: Addition recovery rate (%) = measured value/added value × 100%; coefficient of variation (%) = standard error (SD)/average value × 100%. The UPLC–MS/MS method was used to verify the results of the ic-ELISA-based nanobody detection. The conditions were used as follows: mobile phase A consisted of 5 mmol/L ammonium formate and 0.1% formic acid in methanol, and mobile phase B consisted of 5 mmol/L ammonium formate and 0.1% formic acid in water. The gradient elution was 0–9 min, 90% B-50% B; 9–20 min, 50% B-35% B; 20–29 min, 35% B-0; and 30–34 min, 0–90% B. The flow rate of the mobile phase was 0.3 mL/min, and 10 μL of each sample was injected into the UPLC system.

## 3. Results and Discussion

### 3.1. Library Construction and Selection of Anti-Carbofuran Phage Clones

**Figure 1:**
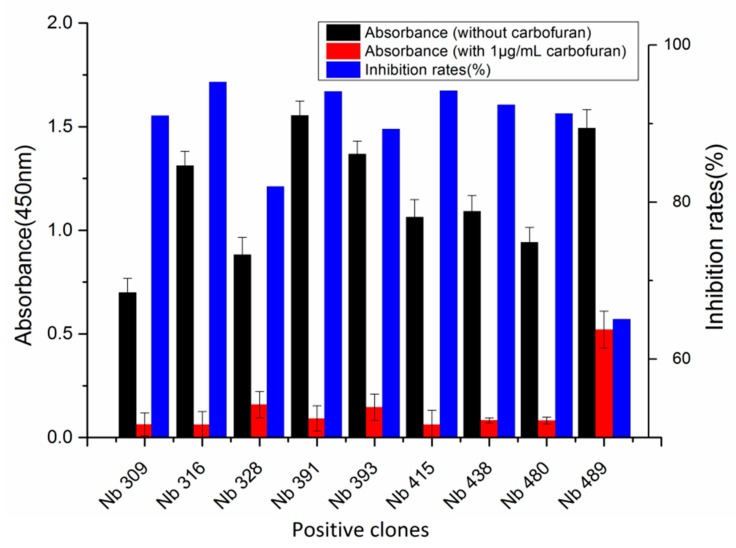
Nine phage clones binding to carbofuran selected and identified by ic-ELISA. Inhibition rate = (absorbance at 450 nm without carbofuran—absorbance at 450 nm with 1 μg/mL carbofuran)/absorbance at 450 nm without carbofuran. Nb316 showed the highest inhibition rate for carbofuran.

**Figure 2:**
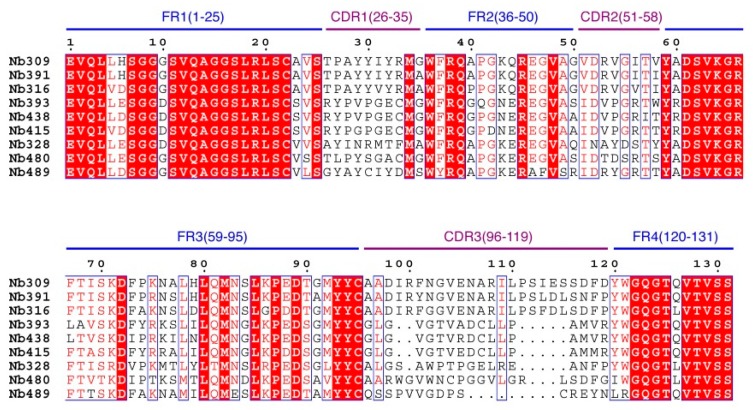
Sequence alignment of nine positive clones.

A phage display nanobody library was obtained via genetic recombination and the calculated size of the library was 1.06 × 10^8^ cfu/mL. Twenty-five clones that randomly picked from LB plates were sequenced, and the diversity of library sequence was 100%. After rescuing with 10^12^ pfu/mL helper phage M13K07, the phage display nanobody library titer was 10^11^ pfu/mL. After the second round, the phage output increased from 10^5^ to 10^7^ pfu/mL, indicating significant enrichment of specific phage clones binding to the coated antigen BFNB-OVA. The third and fourth output was selected to identify specific phage clones. The results showed that 80% of all the clones bound to BFNB-OVA and differed in their binding activity to carbofuran, indicating efficient experimental screening. All positive clones with inhibition rates (with 1 μg/mL carbofuran) greater than 50% were sequenced, and nine groups of clones were classified based on the complementarity determining region 3 (CDR3). Clones with the highest inhibition rate in each group were selected: Nb309 (393 bp), Nb316 (393 bp), Nb328 (375 bp), Nb391 (393 bp), Nb393 (372 bp), Nb415 (372 bp), Nb438 (372 bp), Nb480 (384 bp), and Nb489 (366 bp). The results of ic-ELISA (Figure 1) showed that the inhibition rates of nine clones with 1 μg/mL carbofuran were 91.0%, 95.3%, 82.0%, 94.1%, 89.3%, 94.2%, 92.4%, 91.3%, and 65.1%, respectively. Nine clone sequences were aligned, and the sequence similarity of the framework regions in the nine clones was extremely high and very conserved (Figure 2). The CDRs, especially CDR3 (15–24 amino acids), showed rich diversity. Among these nine clones, Nb316 showed the highest inhibition rate and was used for subsequent studies.

### 3.2. Preparation and Stability Analysis of Anti-Carbofuran Nanobody 

**Figure 3:**
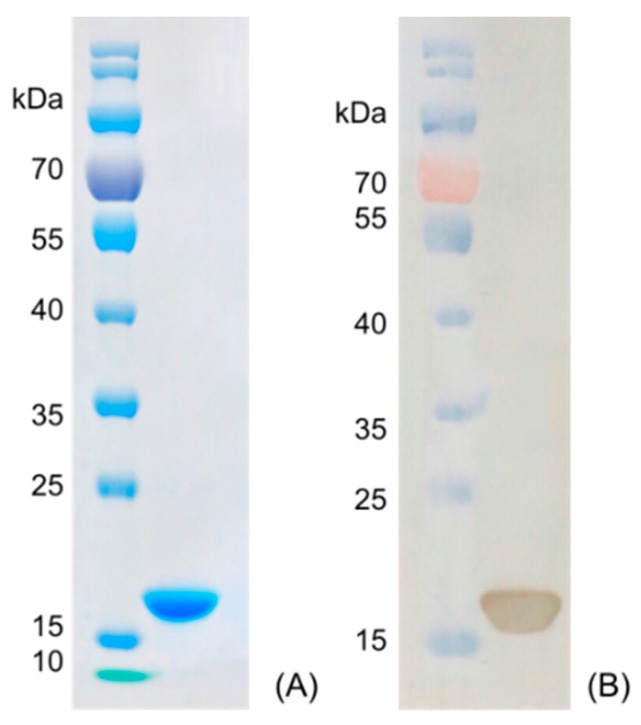
Characterization of nanobody Nb316 by SDS-PAGE and Western blotting. (**A**) SDS-PAGE: marker and purified Nb316. (**B**) Western blotting: marker and purified Nb316.

**Figure 4:**
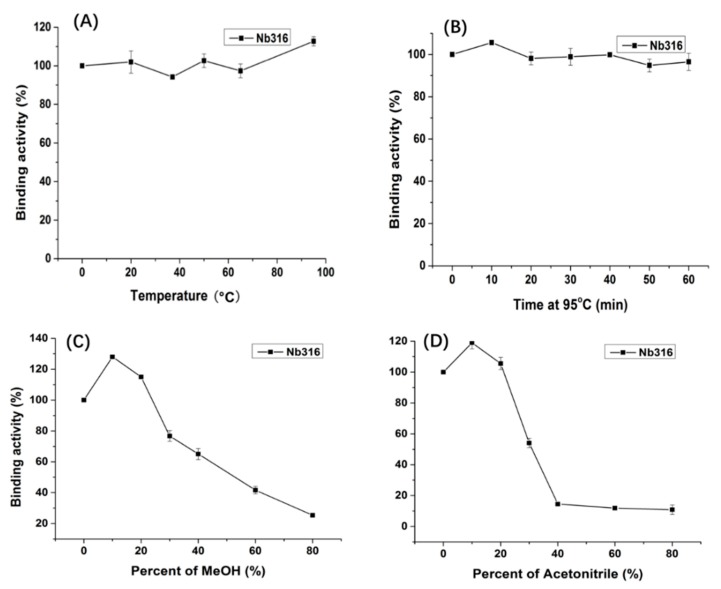
Thermostability and organic solvents tolerance of Nb316 by indirect ELISA based on the antigen BFNB-OVA and anti-HA-HRP antibody. (**A**) Nb316 (1 mg/mL) was incubated at 20, 35, 50, 65, 80, and 95 °C for 5 min; (**B**) Nb316 (1 mg/mL) was incubated at 95 °C for 10, 20, 30, 40, 50, and 60 min. A series concentration (10%, 20%, 40%, 60%, and 80%) of (**C**) MeOH and (**D**) acetonitrile was as the dilution reagents with Nb316.

Nanobody Nb316 was expressed in *Escherichia coli* BL21 (DE3) and then purified (Figure 3). The protein yield was about 2 mg/L and the purity was higher than 90%. The stability of nanobody was evaluated by the antigen-binding activity (the binding ability between antigen and antibody) at high temperature and in the presence of high concentration of organic solvents. After incubation for 5 min at different temperatures, the binding activity of Nb316 was not substantially reduced. Moreover, the binding activity remained at about 100% during incubation for 1 h at 95 °C (Figure 4A,B). The high thermal stability of nanobodies may be attributed to four hallmark nanobody-specific amino acids, Phe, Glx, Arg, and Gly in framework region 2 (FR2), which increase the hydrophilicity of the antibody and stabilize nanobodies [26,27]. In addition, the heat resistance of nanobodies is also related to reversible refolding, as well as the amount and location of disulfide bonds [28,29,30,31].

Immunoassays are usually carried out in aqueous solution, which is an ideal environment for antibodies. However, some environmental analytes, such as pesticides, are not easily dissolved in water, and sample extraction requires an organic solvent. Therefore, the high tolerance of antibody to organic solvents is extremely important. The binding activity of the nanobody Nb316 to the coating antigen is promoted in 10% methanol and acetonitrile (Figure 4C,D). The nanobody still shows 50% binding activity when the methanol concentration is 50% and the acetonitrile concentration is 30%. Thus, nanobodies also exhibit high tolerance to high concentrations of methanol and acetonitrile. The excellent thermal stability and tolerance of nanobodies to organic solvents promoted the stability of nanobodies in actual test samples.

### 3.3. Nanobody-Based Ic-ELISA

**Figure 5:**
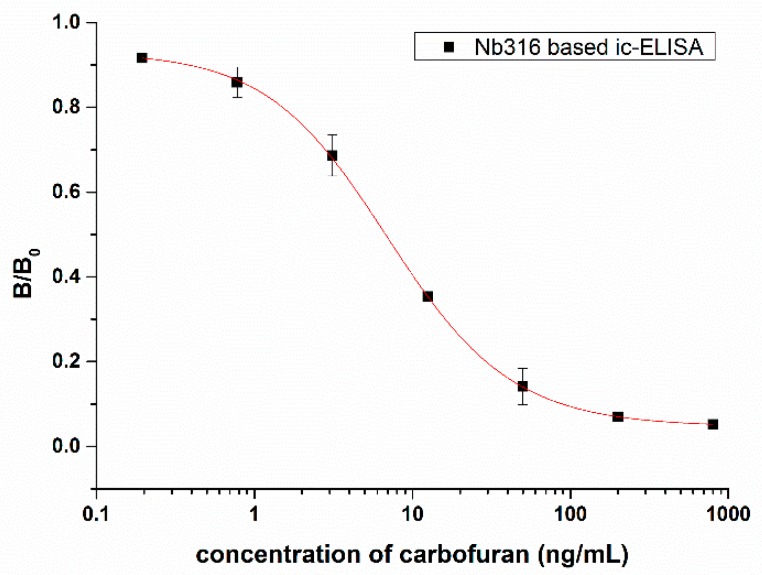
Standard competitive inhibition curve for carbofuran analysis under the optimized conditions.

An ic-ELISA method based on Nb316 for the detection of carbofuran was established. The coating antigen and nanobody concentration were optimized via checkerboard titration. The optimal concentration of coating antigen is 1 μg/mL and nanobody is 4 μg/mL. As Figure 5 shows, the IC_50_ was 7.27 ± 0.88 ng/mL. The linear range varied from 1.44 ng/mL to 30.39 ng/mL, and the detection limit was 0.65 ± 0.24 ng/mL. 

The specificity of nanobody was determined by comparing with IC_50_ values of other standard curves of carbofuran structural analogs. The IC_50_ values ic-ELISA based on Nb316 to benfuracarb, fenobucarb, carbosulfan, 3-hydroxycarbofuran, and isoprocarb was 142.51, 204.6, 280.93, 366.05, and 1351.82 ng/mL, with cross-reactivities (CRs) of 5.1%, 3.5%, 2.6%, 2.0%, and 0.5%, respectively (Table 1). The CRs of seven other structural analogs were all below 0.1%. The nanobody Nb316 showed good sensitivity and specificity for carbofuran recognition. Compared with the monoclonal antibody-based ELISA (IC_50_ value, 18.49 ng/mL) previously established by Yang et al. in this laboratory, the method adopted in this study was more sensitive [22]. Also, Zhu et al. used hapten BFNP to develop a direct competitive enzyme-linked immunoassay for carbofuran, and the IC_50_ value was 36.1 ng/mL [32]. The IC_50_ of the method of Moreno et al. is 0.74 ng/mL, but the method is a competitive heterologous ELISA in the antibody-coated format [33].

### 3.4. Sample Analysis by ic-ELISA and UPLC–MS/MS

**Figure 6:**
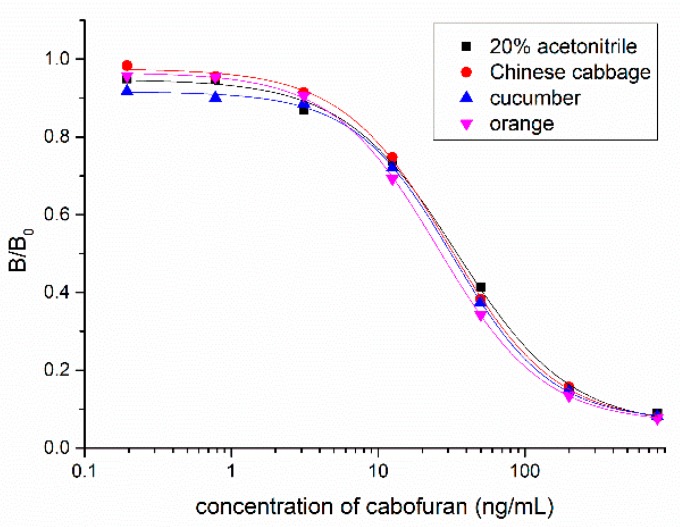
Matrix effects of Chinese cabbage, cucumber, and orange samples.

**Figure 7:**
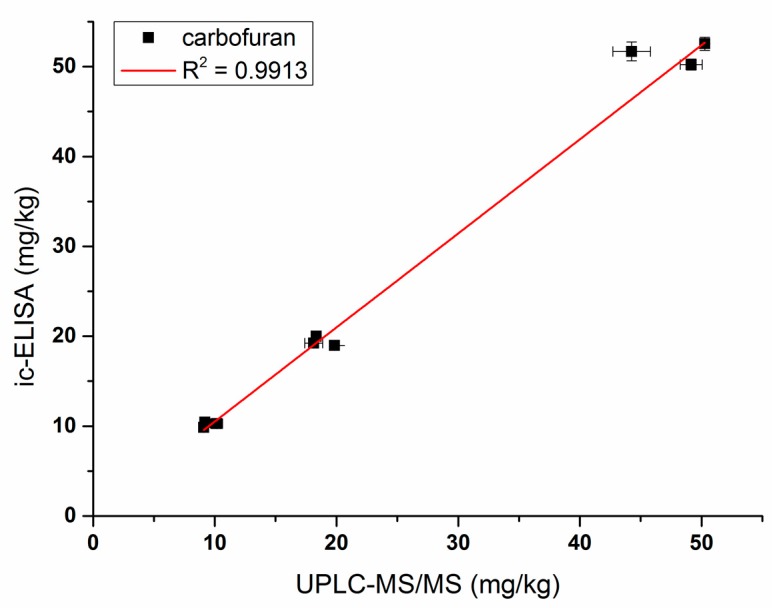
Correlations of analysis of samples spiked with carbofuran between Nb316-based ic-ELISA and UPLC–MS/MS.

Vegetable and fruit samples were extracted with acetonitrile. The matrix effect was tested with different dilutions and it was found that the matrix effect could be eliminated with a five-fold PBS dilution. The ic-ELISA standard curve established with the five-fold blank sample extract was substantially identical to the standard curve established with 20% acetonitrile buffer (Figure 6). Vegetable and fruit samples spiked with different concentrations (10, 20, and 50 ng/g) of carbofuran were measured by ic-ELISA (Table 2). The average recovery of samples varied between 82.3% and 103.9% following the addition level. The coefficient of variation was within 10%, which met the testing requirements of actual samples. To evaluate the accuracy of ic-ELISA method, the fortified samples were also validated by UPLC–MS/MS. The correlation coefficient between ic-ELISA and UPLC–MS/MS was 0.9913 (Figure 7). Overall, the pretreatment method omitting the evaporation step of organic solvent is feasible, and Nb316-based ic-ELISA method can be used for the detection of carbofuran in vegetables and fruits accurately and reproducibly.

## 4. Conclusions

A nanobody Nb316 specific for carbofuran with good thermal stability and high resistance to methanol and acetonitrile was isolated from the phage display library. Based on the nanobody, a competitive indirect ELISA was developed and used to determine carbofuran in vegetable and fruit samples. Simple pretreatment omitting evaporation step of organic solvent was used. The proposed immunoassay showed good sensitivity and specificity to carbofuran. The proposed methods validated by standard UPLC–MS/MS were accurate and reproducible. A nanobody-based immunoassay is an ideal screening tool for carbofuran monitoring due to its simplicity, rapidity, and cost-effectiveness.

## Figures and Tables

**Table 1:** Cross-reactivity of nanobody Nb316 with carbofuran structural analogues.

Analogues	Molecular Structural	IC_50_ (ng/mL)	Cross-Reactivity (%)
Carbofuran	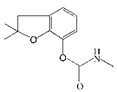	7.27	100
Benfuracarb	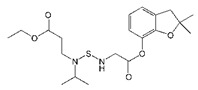	142.51	5.1
Fenobucarb	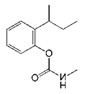	204.6	3.5
Carbosulfan	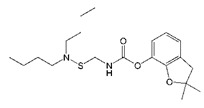	280.93	2.6
3-Hydroxycarbofuran	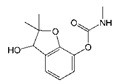	366.05	2.0
Isoprocarb	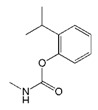	1351.82	0.5
Carbaryl	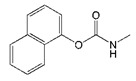	>2000	<0.1
Aldicarb	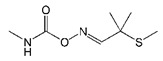	>2000	<0.1
Methomyl	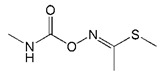	>2000	<0.1
Pirimicarb	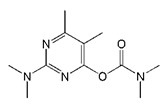	>2000	<0.1
Mercaptodimethur	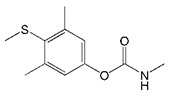	>2000	<0.1
Tsumacide	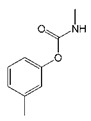	>2000	<0.1

**Table 2:** Recovery analysis of carbofuran in Chinese cabbage, cucumber, and orange samples by ic-ELISA.

Added (mg/kg)	Chinese Cabbage			Cucumber			Orange		
	Found ± SD (mg/kg)	Recovery (%)	CV (%)	Found ± SD (mg/kg)	Recovery (%)	CV (%)	Found ± SD (mg/kg)	Recovery (%)	CV (%)
10	10.27 ± 0.34	102.7	3.33	9.73 ± 0.24	97.35	2.52	8.47 ± 0.2	84.74	2.35
20	18.44 ± 0.54	92.25	2.61	20.45 ± 0.31	102.26	1.50	16.46 ± 0.22	82.31	1.21
50	51.96 ± 0.75	103.92	1.44	46.24 ± 0.51	92.48	1.11	43.77 ± 0.91	87.54	20.76
